# 100 Days of marine *Synechococcus*–*Ruegeria pomeroyi* interaction: A detailed analysis of the exoproteome

**DOI:** 10.1111/1462-2920.14012

**Published:** 2017-12-15

**Authors:** Amandeep Kaur, Juan R. Hernandez‐Fernaud, Maria del Mar Aguilo‐Ferretjans, Elizabeth M. Wellington, Joseph A. Christie‐Oleza

**Affiliations:** ^1^ School of Life Sciences University of Warwick Coventry CV4 7AL UK

## Abstract

Marine phototroph and heterotroph interactions are vital in maintaining the nutrient balance in the oceans as essential nutrients need to be rapidly cycled before sinking to aphotic layers. The aim of this study was to highlight the molecular mechanisms that drive these interactions. For this, we generated a detailed exoproteomic time‐course analysis of a 100‐day co‐culture between the model marine picocyanobacterium *Synechococcus* sp. WH7803 and the *Roseobacter* strain *Ruegeria pomeroyi* DSS‐3, both in nutrient‐enriched and natural oligotrophic seawater. The proteomic data showed a transition between the initial growth phase and stable‐state phase that, in the case of the heterotroph, was caused by a switch in motility attributed to organic matter availability. The phototroph adapted to seawater oligotrophy by reducing its selective leakiness, increasing the acquisition of essential nutrients and secreting conserved proteins of unknown function. We also report a surprisingly high abundance of extracellular superoxide dismutase produced by *Synechococcus* and a dynamic secretion of potential hydrolytic enzyme candidates used by the heterotroph to cleave organic groups and hydrolase polymeric organic matter produced by the cyanobacterium. The time course dataset we present here will become a reference for understanding the molecular processes underpinning marine phototroph‐heterotroph interactions.

## Introduction

The ocean is the Earth's largest biome covering 70% of the world's surface. Marine systems play a major role in global climate regulation, not only due to their ability to store and transport heat, but also because of the constant atmosphere—ocean exchange of CO_2_. Oceans are major carbon reservoirs and are known to buffer anthropogenic carbon emissions by drawing CO_2_ from the atmosphere and burying it in the deep ocean, a process known as the biological carbon pump (Jiao and Zheng, 2011; De La Rocha and Passow, 2014). This is a process whereby CO_2_ in the upper ocean is fixed by photosynthetic primary producers to form organic matter that is then transported to the deeper ocean by sedimenting particulate organic matter (POM) and the drawdown of dissolved organic matter (DOM) through mixing and dwelling (Jiao *et al*., 2010). Marine microbes are also major drivers of other global biogeochemical cycles such as those of nitrogen and sulphur (Karl and Church, 2014).

Marine picocyanobacteria, mainly belonging to the genera *Prochlorococcus* and *Synechococcus*, are numerically the world's dominant photosynthetic primary producers and a major component of marine phytoplankton. Despite being outnumbered by the heterotrophic community, these cyanobacteria are responsible for half of the marine primary production and play a key role in sustaining marine food webs (Falkowski, 2012). Hence, phytoplankton are at the base of the marine food chain feeding the ecosystem with DOM and POM that is released via cell lysis (e.g., cell death, inefficient grazing and viral lysis) or other cellular processes (e.g., outer membrane vesicles, active efflux processes or permeable membrane leakage) (Biller *et al*., 2014; Christie‐Oleza *et al*., 2015a, 2017; Grossowicz *et al*., 2017). Most of this organic matter will be used by the heterotrophic bacterioplankton as their main source of carbon and energy, returning inorganic nutrients to the phototrophic community (Christie‐Oleza *et al*., 2017). The *Roseobacter* group of the class Alphaproteobacteria is an important component of the microbial community within the marine euphotic layer, accounting for up to 20% of the sea surface bacterioplankton (Wagner‐Dobler and Biebl, 2006). This group of heterotrophs generally shows a positive correlation with the phytoplankton community and forms intimate associations with specific phytoplankton groups (Giebel *et al*., 2011; Morris *et al*., 2012). Interestingly, marine *Roseobacter* strains present a large genomic capability and metabolic versatility to use an array of organic substrates found within phytoplankton exudates and, hence, they are one of the first bacterioplankton groups to react to the input of organic matter produced, for example, during phytoplankton blooms (Newton *et al*., 2010; Romera‐Castillo *et al*., 2011; Christie‐Oleza *et al*., 2012a; Buchan *et al*., 2014; Landa *et al*., 2016; Simon *et al*., 2017). The strain *Ruegeria pomeroyi* DSS‐3 was the first roseobacterium to have its genome sequenced (Moran *et al*., 2004) and has served as a model organism to study biogeochemical, ecological and physiological strategies of this group of heterotrophic marine bacteria (Christie‐Oleza and Armengaud, 2015).

Around 70% of the DOM in the oceans is considered of low‐molecular weight (< 1 kDa) (Benner, 2002). The other 30% of DOM is of high molecular weight (> 1 kDa) and, curiously, is much less refractory and, hence, more readily degraded than the low‐molecular weight fraction (Decho and Gutierrez, 2017). Most of the high molecular weight DOM is in the form of biopolymers, and because biological membrane systems are only permeable to molecules smaller than 0.6 kDa (Weiss *et al*., 1991), exoenzymes or ectoenzymes play a key pivotal role in polymeric DOM hydrolysis and assimilation (Vetter and Deming, 1999) as these biopolymers must be hydrolyzed outside the cell before they can be taken up by most organisms (i.e., nongrazing organisms). The activities of secreted enzymes in marine microbes has generally been assessed through the use of fluorescently labeled substrates (Karner and Herndl, 1992; Martinez *et al*., 1996; D'Ambrosio *et al*., 2014; Arnosti, 2015), simple observation of bacterial growth on different polymeric substrates (e.g., Mitulla *et al*., 2016) or, more rarely, identifying and characterizing the actual enzymes involved in hydrolyzing the DOM (e.g., Hehemann *et al*., 2014; Xing *et al*., 2015). Interestingly, marine bacterial exoenzymes are proving highly distinct from their well‐characterized terrestrial counterparts (Michel and Czjzek, 2013) and are currently poorly identified.

The use of shotgun proteomics has become a powerful tool for detecting the array of proteins present in the extracellular medium of an organism under different experimental conditions (Armengaud *et al*., 2012) and, hence, a reliable high throughput method for identifying key secreted enzymes and proteins involved in cell‐to‐cell and cell–environment interactions. We previously analyzed the exoproteome of various *Roseobacter* strains grown in rich media and showed a diversity of trophic strategies within this clade, that is, through the abundant detection of secreted nutrient transporters, mobility proteins, adhesion‐like proteins or toxins (Christie‐Oleza *et al*., 2012b). Nevertheless, *Roseobacter* strains induced a completely different array of secreted proteins when grown in the presence of a more realistic source of organic matter, that is, DOM and POM produced by marine *Synechococcus*, with an increase in proteins involved in motility, microbial interactions, hydrolytic activities and capsid proteins of the genetic transfer agent (GTA) encoded in the genome and a strong decrease in the secretion of toxin‐like proteins (Christie‐Oleza *et al*., 2015b).

*Synechococcus* species generate large amounts of organic matter and requires the presence of a specialized heterotrophic community to remineralise the leaked photosynthate and obtain a constant feed‐back of inorganic nutrients (Christie‐Oleza *et al*., 2017). Based on this basic principle of phototroph–heterotroph interaction, illuminated *R. pomeroyi*–*Synechococcus* sp. co‐cultures are able to survive for extended time periods both in mineral enriched media and natural oligotrophic seawater (Christie‐Oleza *et al*., 2017). In this study, we analyzed for the first time the exoproteome of *R. pomeroyi*–*Synechococcus* co‐cultures under an extended time period (i.e., 100 days) both in nutrient‐enriched and natural oligotrophic seawater in order to generate a unique time course dataset that would highlight the molecular mechanisms involved in this dependent microbial interaction over time. We therefore aimed to (i) determine protein pattern shifts over the 100‐day time course and observe whether culture stability is reflected by a consistent exoproteome; (ii) identify culture stages and frame the trophic strategy of each microbe at each time point; and (iii) identify the secreted hydrolytic enzymes produced by the heterotroph and evaluate their variations over time.

## Results and discussion

### Correlation between culture growth and exoproteomes

**Figure 1:**
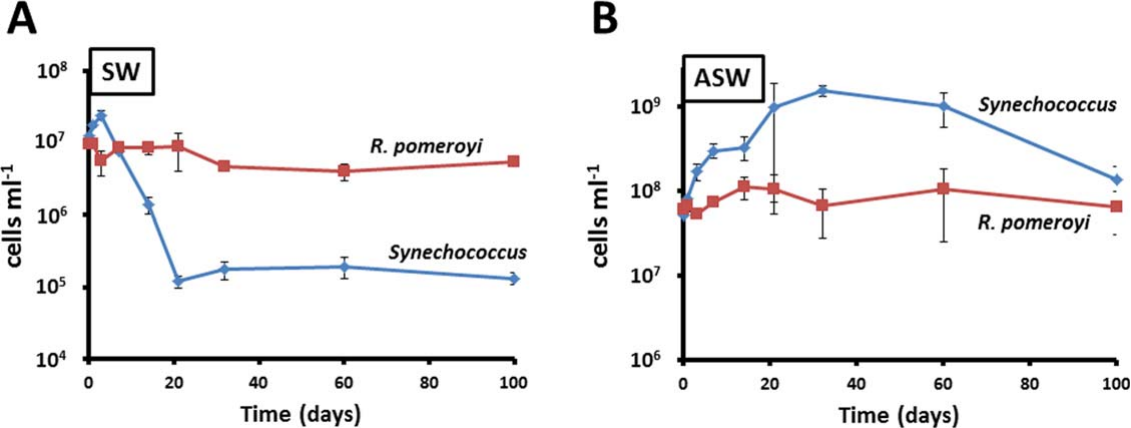
Growth curves of *R. pomeroyi* DSS‐3 and *Synechococcus* WH7803 over the 100‐day time course experiment in natural SW (A) and ASW medium (B). The average value of triplicate cultures (*n* = 3) is shown in panels (error bars show standard deviation).

In both nutrient‐rich and oligotrophic media, the sustained survival of the co‐culture comes as a consequence of nutrient cycling between the phototroph and heterotroph (Christie‐Oleza *et al*., 2017). Here, the growth of *Synechococcus* sp. WH7803 and *R. pomeroyi* DSS‐3 during the 100 days co‐culture in both natural oligotrophic seawater (SW) and enriched artificial seawater (ASW) (Fig. 1) showed the expected trends as previously reported (Christie‐Oleza *et al*., 2017). While *Synechococcus* sp. WH7803 reaches high cell densities in ASW (> 10^9^ cells ml^−1^) and growth is limited by light, in natural SW the cyanobacterium is limited by the availability of inorganic nutrients and cell densities reached 10^5^ cells ml^−1^, numbers that are similar to those observed in natural marine ecosystems (Parsons *et al*., 2012). The heterotroph, *R. pomeroyi* DSS‐3, is limited by the availability of organic carbon and vitamins regenerating essential nutrients for the phototroph. *Synechococcus*–*R. pomeroyi* co‐cultures can persist over 200 days (Christie‐Oleza *et al*., 2017), but a sTable state is reached after just 21 days in natural SW (Fig. 1). However, in rich ASW medium, *Synechococcus* sp. WH7803 only enters the sTable state after 100 days when cell densities drop 10‐fold and stabilise at 10^8^ cells ml^−1^ (Christie‐Oleza *et al*., 2017).

**Figure 2:**
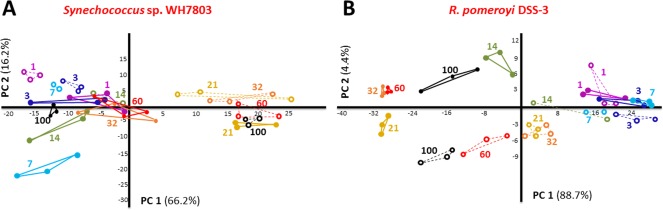
PCA of the normalized exoproteomes of *Synechococcus* sp. WH7803 (A) and *R. pomeroyi* DSS‐3 (B) when grown in co‐culture in ASW medium (solid circles and lines) and natural SW (open circles and dashed lines). Numbers refer to the culture day that the samples were collected.

Exoproteomes tend to reflect microbial adaptive strategies (Christie‐Oleza *et al*., 2012bb), and as expected, the variations observed in the exoproteomes presented here corresponded nicely with the different growth‐phase physiologies observed in SW and ASW grown co‐cultures as shown by the principal component analyses (PCA) using the normalized exoproteomic data obtained from *Synechococcus* sp. WH7803 and *R. pomeroyi* over the time course experiment. The sum of the two first principal components represented 82% and 93% of the variability within the exoproteome of *Synechococcus* sp. WH7803 and *R. pomeroyi* over the 100 days, respectively (Fig. 2).

#### PCA of the exoproteome of *Synechococcus* sp. WH7803

In natural SW, the exoproteome of *Synechococcus* showed a distinct shift between time points 1–14 and 21–100 days incubation (Fig. 2A), which coincides with the co‐culture entering the sTable state equilibrium observed at day 21 (Fig. 1). Interestingly, the exoproteome of ASW‐grown *Synechococcus* sp. WH7803 remained similar over the 100 days experiment, except for time point 21 which marks the transition from the exponential to the sTable state phase (Fig. 1). Curiously, time point 21 grouped with the sTable state time points of *Synechococcus* grown in oligotrophic SW (i.e., 21–100 days; Fig. 2A), suggesting a transitory peak of starvation in ASW media.

#### PCA of the exoproteome of *R. pomeroyi*

The heterotroph showed a similar transition over time in both nutrient‐poor and ‐rich media (Fig. 2B), mainly marked by the shift from exponential and sTable state cultures. Nevertheless, the slight divergence between SW and ASW exoproteomes was mainly resolved by the principal component 2 (Fig. 2B), suggesting a distinctness between nutrient‐rich and oligotrophic culture conditions.

Although the transitions that were observed over time are likely caused by the acclimation of the microbial populations to the varying conditions of the culture, genetic modifications (as demonstrated in Zambrano *et al*., 1993) cannot be ruled out and, hence, further research is needed to determine if genetic evolution also occurs during these long‐term co‐cultures.

### The exoproteome of *Synechococcus* over the 100‐day time course

**Figure 3:**
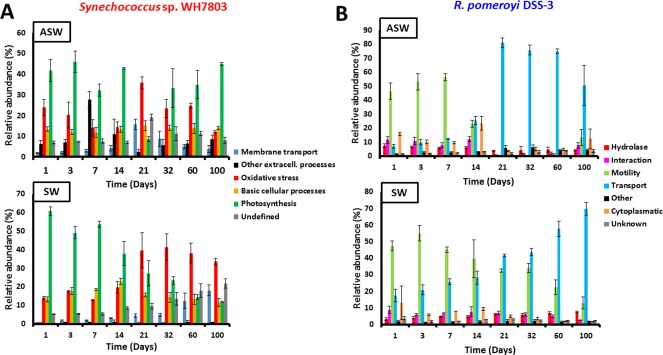
Functional category abundance of protein found in the exoproteomes of *Synechococcus* sp. WH7803 (A) and *R. pomeroyi* DSS‐3 (B) when grown in co‐culture in ASW medium and natural SW. The average value of triplicate cultures analyses (n = 3) are shown (error bars show standard deviation).

Just 187 and 221 of the 681 polypeptides detected in the co‐culture's exoproteome belonging to *Synechococcus* already represented 95% of the total protein abundance in SW and ASW, respectively. These proteins were grouped into functional categories (Supporting Information Table S4) and represented in Figure 3A.

#### Photosynthesis and basic cellular processes

*Synechococcus* sp. release considerable amounts of organic matter not only in the form of carbohydrates (Biersmith and Benner, 1998; Bertlisson *et al*., 2005) but also in the form of protein (Christie‐Oleza *et al*., 2015a; 2017). Here, proteins involved in photosynthesis are among the most abundant categories found in the exoproteome of *Synechococcus* sp. WH7803, representing around 32% of the exoproteome (Table 1). Interestingly, this percentage is strikingly similar to the amount of photosynthetic proteins observed in cellular proteomic analyses (Table 1; Christie‐Oleza *et al*., 2017). While this may suggest that the exoproteome of *Synechococcus* is made entirely of proteins generated from cell lysis, other indicators such as the amount of cytoplasmic or ribosomal proteins are not in agreement. While ribosomal proteins in *Synechococcus* cells represent between 5.5% and 6.5% of the total cellular proteome, only between 1.0% and 1.6% of relative abundance of these proteins are found in the extracellular fraction (Table 1), suggesting that between 18% and 25% of the exoproteome may well come from cell lysis. When using cytoplasmic proteins as an indicator, the average percentage of predicted cell lysis increases to 50% (Table 1), but with bursts that coincide with the cell lysis expected from the growth curves. For example, the drastic reduction of *Synechococcus* cell abundance observed at days 7 and 14 under SW conditions (Fig. 1A) coincides with an increase in detection of cytoplasmic proteins in the exoproteome (i.e., 25–30%), suggesting that, during these time points, between 65% and 77% of the extracellular proteins may come from cell lysis. Nevertheless, at other time points cell lysis remains stably between 40% and 50%. It is interesting to note that some of these predicted cytoplasmic proteins are more abundant in the exoproteome than in the cellular fraction suggesting one of the following three possibilities: (i) they are more sTable in the extracellular milieu than other cytoplasmic proteins and, therefore, over time they are enriched in the exoproteome; (ii) they might actually be actively secreted; and (iii) they are ‘selectively leaked’ as suggested by others (Grossowicz *et al*., 2017). One of these proteins is the nucleotide‐binding protein SynWH7803_1823, a protein predicted to be involved in the temporal control of bacteriophage gene transcription, which represents 2.4% of *Synechococcus* exoproteome (almost 12% of the predicted cytoplasmic proteins in this fraction), whereas it only represents 0.1% in the cellular proteome. Proteins involved in photosynthesis are another example of unequal accumulation in the extracellular milieu or ‘selective leakiness’, and different elements of the photosynthetic apparatus are differentially partitioned, such as those from the phycobilisome (Table 1). Phycobilisomes should be localized on the cytoplasmic side of the thylakoidal membrane, but they seem to be enriched in the exoproteome of *Synechococcus*. Interestingly, the presence of these proteins from the photosynthetic antenna shows a strong decrease in the natural SW exoproteome over time (from 55% to 5% of the exoproteome; Fig. 3A and Supporting Information Table S4), suggesting a reduction in leakiness or a decrease in the production of cellular phycobilisomes as a consequence of nutrient stress. Protein trafficking in cyanobacteria remains poorly characterized (Schneider, 2014) and the ‘selective leakage’ observed here, such as of predicted cytoplasmic proteins or phycobilisomes, requires further research.

**Table 1:** Relative abundance of ribosomal proteins and proteins form the photosynthetic apparatus detected in *Synechococcus* sp. WH7803 proteome datasets.

	Cellular fraction^a^		Exoproteome fraction^b^			
	SW	ASW	SW		ASW	
*Synechococcus*						
Cytoplasmic proteins^c^	38.5 ± 1.1	33.1 ± 1.0	19.4 ± 4.9	50%	16.6 ± 2.1	50%
Ribosomal proteins	6.5 ± 0.9	5.5 ± 1.0	1.6 ± 0.8	25%	1.0 ± 0.6	18%
Photosynthetic apparatus	35.9 ± 1.3	39.4 ± 1.8	31.1 ± 19.8	87%	32.2 ± 11.6	82%
Phycobilisomes	29.8 ± 1.1	28.4 ± 1.6	28.2 ± 19.2	95%	29.2 ± 11.7	103%
Other elements	6.1 ± 0.4	11.1 ± 0.3	2.9 ± 0.8	48%	2.9 ± 0.7	26%
*R. pomeroyi*						
Cytoplasmic proteins^c^	57.6 ± 1.3	n.a.	6.0 ± 2.5	10%	10.4 ± 6.4	18%
Ribosomal proteins	8.0 ± 0.4	n.a.	0.2 ± 0.2	3%	0.8 ± 0.7	10%

n.a., not applicable due to the low number of proteins detected for *R. pomeroyi* in this analysis.

a Data obtained from cellular proteomes published in Christie‐Oleza and colleagues (2017). Standard deviation from triplicate samples is shown.

b This study. Standard deviation from triplicate experiments from all eight sampled time points is shown. Percentages were calculated by dividing the protein relative abundance obtained from exoproteome analyses by those obtained in cellular fractions.

c Cytoplasmic proteins were predicted using the prediction server PSORTb.

#### Membrane transport

The periplasmic‐binding component of ABC transporters is commonly found in microbial exoproteomes (Christie‐Oleza and Armengaud, 2010; Johnson‐Rollings *et al*., 2014) and it has been suggested that some of these proteins could be intentionally translocated from the periplasm to the extracellular space (Giner‐Lamia *et al*., 2016). The detection of membrane transporters is a good indicator of (i) the nutrients that are targeted by each organism in a community (Christie‐Oleza *et al*., 2017) and (ii) an organisms' nutrient stress within the system (Saito *et al*., 2014). As expected, *Synechococcus* sp. WH7803 predominantly targets inorganic nutrients, mainly phosphate and metals such as iron (Supporting Information Table S4). Up to three different periplasmic substrate‐binding proteins for phosphate were detected in the ASW condition, although only one of them (i.e., SynWH7803_1045) was abundantly detected in both enriched and oligotrophic conditions. Interestingly, none of these periplasmic‐binding proteins co‐localizes in the genome with the other components of the ABC transporter for phosphate (i.e., permease and ATPase). *Synechococcus* shows a clear rise in P starvation over time under oligotrophic SW conditions as seen by the progressive increase in abundance of the periplasmic substrate binding protein for phosphate SynWH7803_1045 from 0.7% at day 1 to over 14% after the 100 day incubation (Supporting Information Table S4). A similar trend is observed for porins that are linked to P stress (SynWH7803_0993, 0.1–3.1%; SynWH7803_2236, below 0.1–0.7%) and an outer membrane efflux protein involved in protein secretion which needs further characterization (SynWH7803_2199, below 0.01–0.11%). In nutrient‐enriched ASW media, the cyanobacterium has a peak in all nutrient transport proteins (i.e., P and metals) at day 21, which then drops until day 100 (Fig. 3A), an trend that fits the variability observed in the PCA plot in Figure 2A.

#### Oxidative stress

Apart from nutrient recycling (Christie‐Oleza *et al*., 2017), the scavenging of reactive oxygen species (ROS) is considered a pivotal process in marine cyanobacteria – heterotroph interactions where the cyanobacterium, mostly lacking of catalase, relies on the heterotroph for depleting ROS (Hunken *et al*., 2008; Morris *et al*., 2011, 2008). Nevertheless, while catalase only catalyzes the decomposition of hydrogen peroxide, it is the enzyme superoxide dismutase (SOD) which helps deal with superoxide (O^2–^). SODs are found in all marine cyanobacteria although with different metal cofactors (Scanlan *et al*., 2009). While Ni‐containing SODs are prevalent in catalase‐lacking strains, Fe‐containing SODs prevail in catalase encoding cyanobacteria. Cellular proteomes of *Synechococcus* sp. WH7803 generally show below 1% abundance of this enzyme (Christie‐Oleza *et al*., 2017), but SOD has also been detected in the exoproteomes of different marine *Synechococcus* strains ranging between 0.7% and 3.4% (Christie‐Oleza *et al*., 2015a). SODs are possibly periplasmic‐located enzymes with a non‐canonical secretion system, but may also be ‘unavoidably leaked’ through the outer membrane, reinforcing the idea that ROS scavenging can be a public goods process as suggested by others (Morris *et al*., 2012). In this study, the abundance of the Fe‐containing SOD in the exoproteome of *Synechococcus* sp. WH7803 was surprisingly high throughout the entire time course experiment both in SW and ASW averaging 24% and 19% of total protein abundance, respectively (Supporting Information Table S4), and highlighting the relevance of this enzyme for dealing with ROS. The fact it was detected in both nutrient conditions is an indicator that its abundance is not an artefactual result and its variable abundance in ASW suggests it does not come as a consequence of its accumulation in the milieu. The second SOD encoded by *Synechococcus* sp. WH7803, the copper‐contain protein SynWH7803_0951, was also detected in both SW and ASW exoproteomes with an average detection of 0.19% and 0.03%, respectively.

#### Other extracellular processes

The variable abundance of structural type IV pili proteins in the exoproteome of *Synechococcus* sp. WH7803 is the most remarkable aspect within this functional category (Supporting Information Table S4). In a previous study, we already reported a high abundance of the pili proteins SynWH7803_1795 and SynWH7803_1796 (17.9% and 1.3%, respectively), which was exclusive to this strain (Christie‐Oleza *et al*., 2015a). The time course experiment has allowed us to assign the production of these proteins to nutrient‐rich conditions with a peak of SynWH7803_1795 detection at day 7 (25.7%) and an average detection of 8.1% over the 100 days, whereas the detection was low (< 0.1%) in natural SW conditions. The remarkable ecological and physiological roles of the pili need further characterization although a recent study in *Synechococcus elongatus* suggests this structure may be involved in cell buoyancy (Nagar *et al*., 2017). Interestingly, both alkaline phosphatases were detected in the exoproteome of *Synechococcus* sp. WH7803 but only in low abundance and peaked during day 21 in ASW (Supporting Information Table S4).

#### Secreted proteins of unknown function

**Figure 4:**
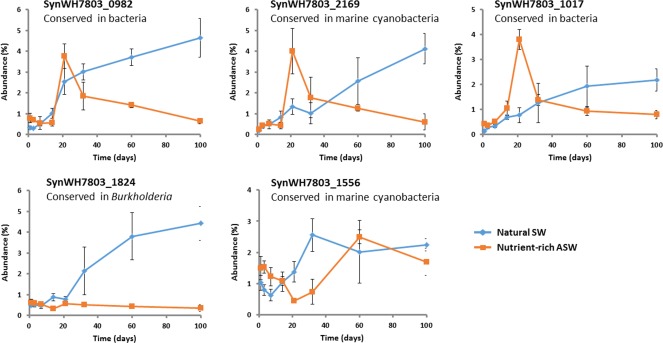
Variation of the five most abundant hypothetical proteins in the exoproteome of *Synechococcus* sp. WH7803 over time. The average value of triplicate cultures analyses (*n* = 3) are shown (error bars show standard deviation).

Proteins of unknown function tend to predominate in the secreted fraction, highlighting the poor knowledge we currently have on microbe–environment interactions (Christie‐Oleza *et al*., 2015a). Datasets such as the one we present here are useful for flagging proteins of unknown function that are abundant and, hence, may have an important role and also to shed light on their possible function by monitoring their abundance over time. Interestingly, the five most abundant proteins in this category were common in both SW and ASW conditions (Supporting Information Table S4), but these showed interesting shifts in abundance over time (Fig. 4). The conserved proteins in bacteria or marine cyanobacteria SynWH7803_0982, SynWH7803_2169 and SynWH7803_1017 showed very similar behavior, with a progressive increase in oligotrophic SW and a peak in abundance at day 21 in ASW (Fig. 4), a time point when *Synechococcus* sp. WH7803 was nutrient‐starved as highlighted above. Hence, these proteins may have a role in response to nutrient stress. In contrast, protein SynWH7803_1824, which is unique to strain WH7803 and conserved only among *Burkholderia*, remained at a constant low abundance but became highly abundant in SW over time (Fig. 4). Finally, protein SynWH7803_1556 showed identical behavior in both culture conditions over time but with a lag of a few days in ASW.

### The exoproteome of *R. pomeroyi* over time

From the 585 proteins detected in the exoproteome of the co‐culture belonging to *R. pomeroyi*, 184 and 222 represented over 95% of the total abundance in SW and ASW, respectively, and were grouped into functional categories (Supporting Information Table S5) and represented in Figure 3B.

#### Cytoplasmic proteins

As mentioned earlier, the detection of predicted cytoplasmic proteins and ribosomal proteins in the exoproteomes can be used as indicators of cell lysis. While the abundance of ribosomal proteins in cellular fractions of *R. pomeroyi* usually represents over 8% of the total proteome (Table 1), here we observed only 0.2% and 0.8% of these proteins in the exoproteomes of SW and ASW co‐cultures, respectively, suggesting that between 3% and 10% of the proteins detected in this study may come from cell lysis. Nevertheless, a higher cell lysis is suggested when predicted cytoplasmic proteins is used as an indicator (i.e., 10–18%; Table 1). There are several reasons that could explain this discrepancy: (i) that dying cells reduce their ribosomal protein content; (ii) the size of ribosomal complexes makes them less ‘leaky’ than other smaller cytoplasmic proteins; and (iii) the prediction of protein trafficking or selective leakiness has been understudied. For example, under both conditions (i.e., SW and ASW), the five most abundant cytoplasmic proteins in the exoproteome of *R. pomeroyi* already contributed to over 50% of the cytoplasmic category, being three of them common between both conditions (i.e., isocitrate dehydrogenase, a hypothetical protein with high similarity to a phosphoenolpyruvate mutase and imidazoleglycerol‐phosphate dehydratase; Supporting Information Table S5), hinting that these proteins, either moonlight (Jeffery, 2009), are dually secreted at least to the periplasm, or are highly sTable in the extracellular milieu and accumulate over time.

#### Motility

**Figure 5:**
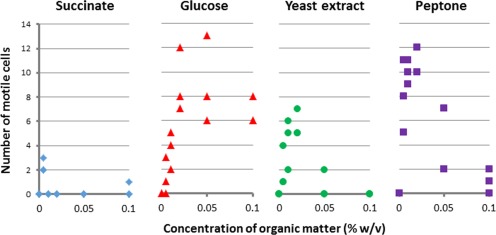
Motility of *R. pomeroyi* DSS‐3 in the presence of varying concentrations of different organic carbon sources. Quantification of motility was based on the number of moving cells observed in 10‐s videos obtained from three different fields per condition.

*Ruegeria pomeroyi* is a flagellum‐propelled motile bacterium. The extracellular flagellin filament and hook are rarely found in the cellular proteome as they are easily sheared off during sample processing and remain in the exoproteomic fraction. The flagellin was detected in very high abundance in the exoproteomes of both SW and ASW conditions (35% and 23%, respectively). During initial culture stages flagellin represented over 50% of the total protein abundance, but while its detection only reduced progressively over time in natural SW (down to 13% at day 100), it dropped to under 1% in ASW between time points 21 and 60 days to finally increase to 13% at day 100 (Fig. 3B). The sharp drop in motility between time points 21 and 60 days in ASW coincides with the maximum *Synechococcus* cell densities (Fig. 1) and, hence, highest photosynthate production and availability. Very low levels of flagellin were also detected in previous studies where *R. pomeroyi* was grown in carbon‐rich media (e.g., the flagellin was not detected in the exoproteome of this strain when grown in marine broth; Christie‐Oleza and Armengaud, 2010; Christie‐Oleza *et al*., 2012b), highlighting the ability of *R. pomeroyi* to switch between a motile–nonmotile lifestyle, which is dependent on nutrient availability. In order to prove this hypothesis, we visualized *R. pomeroyi*'s motility under different nutrient conditions. No cell motility was observed when this strain was incubated in nutrient‐rich marine broth (containing 0.6%, wt/vol, of organic carbon, of which 0.5% is peptone and 0.1% yeast extract) or in carbon depleted media (i.e., ASW or ASW supplemented with ammonium and vitamins). Interestingly, *R. pomeroyi* did show notorious motility when incubated in filter‐sterilized ASW media obtained from a 2‐week old *Synechococcus* culture (Supporting Information Movie S1), which we predict contained ∼ 0.02% (wt/vol) of organic carbon (i.e., mainly protein) based on previous measurements (Christie‐Oleza *et al*., 2017). We then tested the motility of *R. pomeroyi* in the presence of varying concentrations of four different sources of organic matter (Fig. 5 and Supporting Information Movies S2 and S3). Glucose, yeast extract and peptone induced cell motility at concentrations as low as 0.005% (wt/vol). In contrast, higher substrate concentrations of yeast extract and peptone (i.e., 0.1%) caused a drop in motility (Fig. 5), suggesting a substrate repression in flagella biosynthesis as reported previously in other heterotrophic bacteria (Stella *et al*., 2008). These results are in agreement with the proteomic detection of flagellin when *R. pomeroyi* was grown in the presence of *Synechococcus* and demonstrates an adaptive life strategy of this strain in response to different levels of organic matter. Hence, *R. pomeroyi* remains immotile in total absence of a carbon source and very low concentrations of organic matter are enough to switch on its motile phenotype. The drop in flagella abundance observed in natural SW incubations (Fig. 3) is possibly caused by the lower concentrations of organic carbon produced by the phototroph under such nutrient‐deplete conditions. Previous measurements of organic matter in such oligotrophic conditions suggest concentrations as low as ∼ 0.0002% (wt/vol). While we did not test such low substrate concentrations in our controlled experiments (Fig. 5), these small amounts of substrate seem to be enough to induce a low level of flagella production and, hence, motility in *R. pomeroyi*. This is also supported by previous observations of 5.0–6.5% flagellin detected in an exoproteomic analysis where this heterotroph was incubated in natural coastal seawater (Christie‐Oleza *et al* 2012b). The abrupt decrease in flagellin abundance observed in nutrient‐enriched ASW conditions (Fig. 3) is more likely caused by elevated organic matter concentrations (i.e., > 0.1%), which represses the biosynthesis of this structure. This may well simulate a phytoplankton bloom where the heterotroph turns off its motility in order to remain within the high concentration patch.

#### Membrane transport

**Figure 6:**
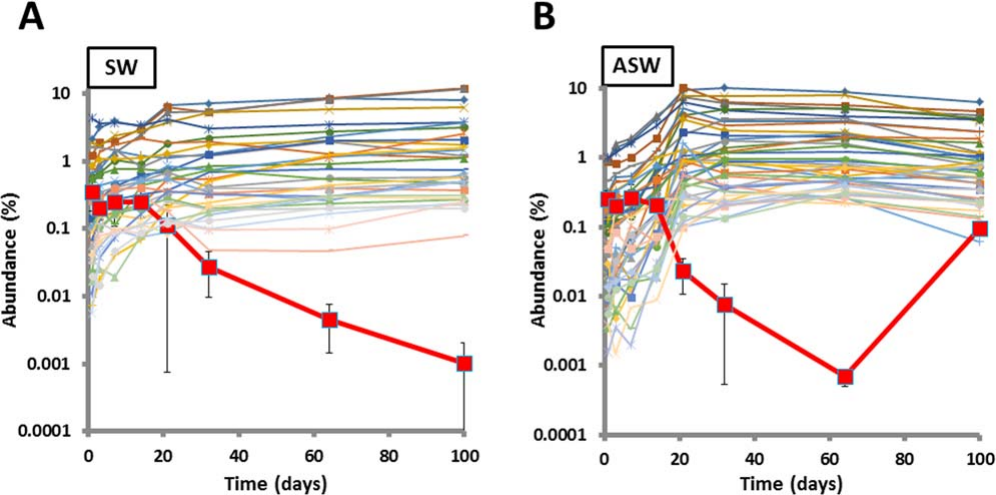
Abundance of *R. pomeroyi* DSS‐3 membrane transport proteins in natural SW (A) and ASW medium (B) over time. Only transporter proteins with average abundance above 0.1% are represented. The average value of triplicate cultures analyses (*n* = 3) are shown. The periplasmic component of the manganese ABC transporter is highlighted in bold and, for convenience, error bars showing standard deviation were added for only this protein.

This is the most abundant category of proteins detected in the exoproteomes of *R. pomeroyi* (42% and 38% in ASW and SW, respectively), mostly made up of outer membrane transport systems and the periplasmic components of inner membrane transporters. For example, the periplasmic substrate‐binding component of ABC transporters made up 100% of the proteins detected for these transport systems, whereas the other components (i.e., permeases and ATP‐binding proteins) remained undetected (Supporting Information Table S5). It is interesting to note that the abundance of membrane transporters mirrors the one of cell motility and highlights how the cell invests resources in response to nutrient availability (Fig. 3B). This generalist marine heterotroph is adapted to scavenge a large variety of scarce nutrients in oligotrophic marine systems and to proliferate rapidly in nutrient‐rich patches of the ocean (Moran *et al*., 2004; Newton *et al*., 2010; Christie‐Oleza *et al*., 2012a, 2015b). The data we present here suggest that this life strategy is achieved by splitting resources between motility to search for nutrients when these are scarce and to rapidly take them up through the investment in membrane transporters when they are abundant. As expected, *R. pomeroyi* induces the production of a large array of specific membrane transporters for importing amino acids, amines and carbohydrates in the presence of *Synechococcus*, which is not surprising as these are the main components of the cyanobacterium photosynthate (Christie‐Oleza *et al*., 2017). Nevertheless, while all transporters showed an increase in abundance in accordance to the pattern shown by the ‘transport’ category in Figure 3B, the periplasmic component of the manganese ABC transporter showed a decrease in abundance in both SW and ASW conditions (Fig. 6). Although it is not clear which specific metal is transported by this transporter (i.e., manganese and zinc), *R. pomeroyi* clearly decreases its uptake possibly favoring the phototroph's high demand in metals for primary production.

#### Interaction

The genetic transfer agent (GTA) and Repeats‐in‐ToXin (RTX‐like) proteins are the main contributors of this functional category. While GTAs are bacteriophage‐like particles produced by a large variety of bacteria which carry random fragments form the host's genome and that are thought to encourage horizontal gene transfer (Frost *et al*., 2005), RTX‐like proteins are elements that mainly play a direct role in cell‐to‐cell interactions ranging from toxicity to adhesion, although due to the enormous extant variability of these proteins, most are of unknown function (Linhartova *et al*., 2010). Interestingly, while RTX‐like proteins were highly produced in organic nutrient‐rich broths, for example, the RTX‐like protein PaxA represented over 50% of the proteins in the exoproteome when *R. pomeroyi* was grown in marine broth (Christie‐Oleza and Armengaud, 2010), this does not occur in the mineral media used in this study, where the heterotroph is constantly supplied with a smaller amount of photosynthate. In any case, RTX‐like proteins were still detected throughout the 100‐day experiment (1.7% and 0.5% in ASW and SW, respectively). GTA are commonly found in *Roseobacter* strains (Biers *et al*., 2008; Zhao *et al*., 2009). The high production of GTA by *R. pomeroyi* when grown in co‐culture with *Synechococcus* was highlighted previously (Christie‐Oleza *et al*., 2015b). However, from the time course data, it is interesting to note that the abundance of GTA elements (with an average protein abundance of 4.6% and 5.7% in ASW and SW, respectively) mimics that of the flagella, that is, dropping from 5% to 9% during days 1–14, to between 0.1% and 0.3% during time points 21–64 days in ASW (Supporting Information Table S5). This suggest a co‐regulation between the motile ‘scavenging‐for‐nutrients’ lifestyle mode and the burst of GTA production, possibly induced by the sensing of low nutrient availability. In fact, GTA production is known to be inhibited by high concentrations of phosphate (Westbye *et al*., 2013).

#### Hydrolytic enzymes for polymeric organic matter

One of the aims of this study was to highlight and monitor the production of potential secreted enzymes involved in the breakdown of phototrophic DOM over time. Marine Alphaproteobacteria are considered to have a very low extracellular hydrolytic potential due to the low number of enzymes that are encoded in their genomes (Barbeyron *et al*., 2016). In fact, previous exoproteomic analyses from a range of *Roseobacter* strains showed that different isolates produced a small but highly divergent, repertoire of exoenzymes suggesting that closely related species may be able to target different substrates and escape competition through the diversification of resources (Christie‐Oleza *et al*., 2015b). Here, the overall average abundance of this category was 5.3% and 5.5% for ASW and SW, respectively, but while the abundance of these proteins progressively dropped in ASW (7.4% at day 1 to 4.1% at day 100), an opposite trend was observed in SW with an increase from 3.3% to 7.6% during the 100‐day time course (Fig. 3B). Nineteen potential hydrolytic enzymes produced by *R. pomeroyi* were detected during this study (i.e., with an average detection > 0.1% in at least one of the culture conditions; Table 2). The two most abundantly detected hydrolases were AAV93776 (2.1% and 2.7% in ASW and SW, respectively) and AAV95476 (0.9% and 2.68% in ASW and SW, respectively), re‐annotated here as a possible pectate lyase and sialidase, respectively (Table 2). Hence, both proteins may be involved in polysaccharide hydrolysis, generating oligosaccharides and short chain sugars that can then be assimilated by the heterotroph. Despite being highly abundant, the pectate lyase‐like protein shows a drastic reduction in the ASW co‐culture between time points 21 and 60 coinciding with the period when *Synechococcus* is most abundant (Fig. 1). During this time period, *Synechococcus* is possibly at its maximum production of photosynthate and *R. pomeroyi* may fill its carbon and energy demands through the use of protein and other organic nitrogen compounds. Interestingly, it is between days 21 and 60 when *R. pomeroyi* shows an increased production of proteases (i.e., AAV95890 and AAV97448) and amidohydrolases (AAV97448 and AAV94260). Members of the *Roseobacter* group are known to preferentially target small nitrogen‐rich DOM (Bryson *et al*., 2016; Teira *et al*., 2017), and hence, it is not surprising that these organisms will specialize in using these compounds when they are present. While the abundance of organic matter in ASW allows *R. pomeroyi* to shift its targeted polymeric DOM, in SW it shows a more sTable production of hydrolases throughout the 100‐day co‐culture, with only the increase of the pectate lyase AAV93776 (from 1.4% to 3.6%) and the protease AAV95890 (from < 0.01% to 0.13%) over time. *Ruegeria pomeroyi* encodes and produces most enzymes required to breakdown the polymeric components within the cyanobacterial photosynthate (i.e., protein, polysaccharides, peptidoglycan and other sulfur and amide compounds), and hence, it is able to mineralize most of the primary produced organic matter, enabling the establishment of long term co‐cultures based on nutrient cycling described previously (Christie‐Oleza *et al*., 2017). Nevertheless, we acknowledge that the identification of most hydrolytic enzymes in Table 2 requires further experimentation to confirm their predicted function. For example, the most abundant enzyme detected, i.e., the pectate lyase‐like protein, was annotated as a hypothetical protein and only suggested here as a hydrolytic enzyme for polysaccharides based on a pectate lyase‐like domain it contains. Other pectate lyases have been detected in marine microbes, but these seem to have been acquired from a terrestrial origin (Hehemann *et al*., 2017). Nevertheless, this is not the norm and most marine microbes have a novel array of untapped hydrolytic enzymes that largely differ from their well‐known terrestrial counterparts (Hehemann *et al*., 2014), which require further characterization.

**Table 2:** Hydrolytic enzymes detected over time in the exoproteome of *R. pomeroyi* DSS‐3 when co‐cultured with *Synechococcus* sp. WH7803.

Locus ID	Annotation (possible function)	Substrate		Day 1^a^	Day 3^a^	Day 7^a^	Day 14^a^	Day 21^a^	Day 32^a^	Day 60^a^	Day 100^a^	Average
AAV93776	Hypothetical SPO0459 (pectate lyase)	Polysaccharides	ASW	4.94	4.05	3.23	3.08	0.04	0.02	0.08	1.63	2.13
			SW	1.43	1.88	2.16	2.29	3.08	3.12	3.84	3.61	2.68
AAV95476	BNR/Asp‐box protein (sialidase)	Polysaccharides	ASW	1.14	1.00	0.99	1.35	0.80	0.43	0.51	1.07	0.91
			SW	1.07	1.08	1.31	1.22	1.77	1.59	1.49	1.25	1.35
AAV95139	Twin‐arginine pathway (phosphatase PhoX)	Phosphates	ASW	0.58	0.56	0.40	0.33	0.16	0.88	0.65	0.15	0.46
			SW	0.53	0.54	0.57	0.28	0.13	0.08	0.02	< 0.01	0.27
AAV96145	Ser/Thr Phosphatase/nucleotidase (nucleotidase)	Nucleotides	ASW	0.04	0.05	0.27	0.13	0.93	0.77	0.70	0.34	0.40
			SW	0.06	0.18	0.23	0.44	0.69	0.58	1.02	1.49	0.59
AAV95890	Protease, S2 family (serine protease)	Proteins	ASW	0.03	0.01	0.01	0.02	0.51	0.56	0.55	0.09	0.22
			SW	< 0.01	0.01	0.01	0.01	0.01	0.03	0.07	0.13	0.03
AAV96272	Metallo‐beta‐lactamase (alkyl sulfatase)	Others	ASW	0.06	0.11	0.12	0.22	0.38	0.26	0.28	0.22	0.21
			SW	0.03	0.10	0.23	0.28	0.35	0.37	0.38	0.43	0.27
AAV93580	Alkaline phosphatase (phosphatase PhoD)	Phosphates	ASW	0.01	0.04	0.03	0.01	< 0.01	0.43	0.78	0.01	0.17
			SW	0.01	0.06	0.04	0.02	0.01	0.02	0.01	< 0.01	0.02
AAV95931	LysM domain/M23/M37 peptidase (peptidase family M23)	Proteins	ASW	0.12	0.28	0.19	0.15	0.11	0.18	0.11	0.10	0.16
			SW	0.05	0.08	0.07	0.06	0.02	0.02	0.01	0.06	0.05
AAV97448	Amidohydrolase protein (amidohydrolase)	Others	ASW	0.04	0.03	0.04	0.04	0.32	0.26	0.27	0.16	0.14
			SW	0.02	0.07	0.11	0.15	0.15	0.08	0.18	0.40	0.14
AAV95236	Beta‐lactamase protein (carboxypeptidase)	Proteins	ASW	0.01	0.05	0.24	0.36	0.03	0.05	0.05	0.09	0.11
			SW	< 0.01	< 0.01	< 0.01	0.01	< 0.01	< 0.01	< 0.01	< 0.01	< 0.01
AAV96841	Peptidoglycan‐binding protein (glycoside hydrolase)	Peptidoglycan	ASW	0.06	0.13	0.07	0.11	0.06	0.10	0.12	0.04	0.09
			SW	0.02	0.09	0.07	0.06	0.03	0.01	0.01	0.03	0.04
AAV94622	Periplasmic serine protease (serine proteases)	Proteins	ASW	0.04	0.05	0.04	0.11	0.06	0.06	0.04	0.03	0.05
			SW	0.01	0.02	0.01	0.04	0.02	0.02	0.01	< 0.01	0.01
AAV95689	Fumarylacetoacetate hydrolase (aromatic hydrolase)	Others	ASW	0.02	0.02	0.05	0.04	0.09	0.07	0.06	0.03	0.05
			SW	0.02	0.03	0.03	0.03	0.02	0.03	0.06	0.05	0.03
AAV96493	Glycosyl hydrolase, family 25 (acetylmuramidase)	Peptidoglycan	ASW	0.08	0.10	0.10	< 0.01	< 0.01	< 0.01	< 0.01	0.01	0.04
			SW	0.03	< 0.01	< 0.01	< 0.01	0.01	< 0.01	< 0.01	< 0.01	0.01
AAV94066	Cyclase family protein (aromatic hydrolase)	Others	ASW	< 0.01	< 0.01	< 0.01	< 0.01	0.07	0.07	0.06	0.04	0.03
			SW	< 0.01	< 0.01	0.01	0.01	0.01	0.02	0.04	0.07	0.02
AAV93452	Murein endopeptidase (murein endopeptidase)	Peptidoglycan	ASW	< 0.01	0.01	0.02	0.02	0.01	0.02	0.08	0.02	0.02
			SW	< 0.01	< 0.01	0.02	< 0.01	< 0.01	< 0.01	< 0.01	< 0.01	0.01
AAV95558	Hypothetical SPO2296 (lysozyme)	Peptidoglycan	ASW	0.02	< 0.01	0.01	0.01	0.01	0.01	0.04	0.03	0.02
			SW	0.02	< 0.01	< 0.01	< 0.01	< 0.01	0.02	< 0.01	< 0.01	0.01
AAV94260	Amidohydrolase protein (amidohydrolase)	Others	ASW	< 0.01	< 0.01	< 0.01	< 0.01	0.04	0.02	0.02	0.01	0.01
			SW	0.01	< 0.01	< 0.01	0.01	< 0.01	< 0.01	< 0.01	< 0.01	< 0.01
AAV96597	Peptidase, M16 family (peptidase M16)	Proteins	ASW	< 0.01	< 0.01	< 0.01	< 0.01	0.03	0.03	0.02	< 0.01	0.01
			SW	< 0.01	< 0.01	< 0.01	0.01	< 0.01	< 0.01	< 0.01	< 0.01	< 0.01

a The values shown are the average of relative abundance obtained from three biological replicate cultures. The full data can be obtained from Supporting Information Table S5.

#### Hydrolytic enzymes for phosphate

Both alkaline phosphatases PhoX and PhoD encoded by *R. pomeroyi* were found in the exoproteomes (Table 2). PhoX, and not PhoA, is prevalent in marine microbes and is thought to play an important role in marine oligotrophic systems (Sebastian and Ammerman, 2009, 2011). PhoX was the predominant alkaline phosphatase in our dataset (Table 2). Nevertheless, the less‐known PhoD, which was in very low abundance throughout most of the time course (abundance below 0.06%), peaked only at time points 32 and 60 (0.43% and 0.78%, respectively) under ASW conditions. While the substrates targeted by PhoX have been characterized (Sebastian and Ammerman, 2011), organic phosphate compounds targeted by PhoD remain unknown. *Ruegeria pomeroyi* also abundantly secretes the nucleotidase AAV96145 when grown in co‐culture with *Synechococcus*. It is worth noting the progressive increase in this nucleotidase especially under SW conditions (from 0.06% to 1.49%), which coincides with a strong decrease in PhoX abundance (Table 2). The ABC transporters for phosphate and organic phosphate (e.g., glycerol‐3‐phosphate) remain constant during the whole time course (Supporting Information Table S5), suggesting that *R. pomeroyi*'s phosphorous starvation status does not vary over time, but the switch from PhoX to a nucleotidase indicates a variation in the organic phosphorous produced by *Synechococcus*. We hypothesize that *Synechococcus* may replace its phospholipids for sulfolipids (Van Mooy *et al*., 2009) and even initiate the accumulation of polyphosphates (Martin *et al*., 2014), which may become, together with nucleic acids, the main source of phosphorous in the system.

## Conclusions

Microbial exoproteomes are good proxies to study the dynamic interactions of microbes with their environment. Here we generated a unique time course exoproteomic dataset of a 100‐day long *Synechococcus* sp. WH7803–*R. pomeroyi* DSS‐3 co‐culture incubated in both nutrient rich and natural oligotrophic seawater. The observed protein variations matched well with the culture's physiology over time and emphasizes the need of time course experiments such as the one we present here in order to obtain a comprehensive understanding of microbial interactions, as single time points may be misleading. This study has highlighted a number of interesting aspects in this phototroph–heterotroph system such as (i) the heterotroph's varying motility lifestyle depending on nutrient availability, (ii) the unexplained selective leakage of phycobilisomes to the milieu by *Synechococcus*, (iii) the specificity of nutrient acquisition though the production of an array of active membrane transport systems, (iv) the large production of SOD by *Synechococcus* to deal with ROS despite the presence of a heterotroph, (v) the varying abundance of a type IV pili structure produced by the phototroph, (vi) a list of uncharacterized hydrolytic enzymes secreted by *R. pomeroyi* to mineralise the polymeric organic matter generated by the cyanobacterium, (vii) a pattern of phosphatases that varies over time and that is believed to adapt to the pool of organic phosphorous present in the system and (viii) a list of relevant proteins of unknown function that require further research. Hence, the high‐resolution time course dataset we present here will become a reference for future characterization of specific molecular mechanisms involved in sustaining this microbial system.

## Experimental procedures

### Bacterial growth and experimental setup

Marine *Synechococcus* sp. WH7803 was grown in ASW (Wilson *et al*., 1996) at 22°C at a light intensity of 10 µmol photons m^−2^ s^−1^ with shaking (140 r.p.m.). *Roseobacter* strain *R. pomeroyi* DSS‐3 was grown in marine broth (Difco, France) at 28°C until stationary phase. *Ruegeria pomeroyi* and *Synechococcus* cells were harvested via centrifugation and washed twice in filter‐sterilized autoclaved seawater (SW, natural seawater collected from the Gulf Stream in the Gulf of Mexico; provided by Sigma, USA) prior to co‐inoculating both organisms in 100 ml ASW and SW at cell concentrations of ∼ 10^8^ and 10^7^ cell ml^−1^, respectively (Fig. 1). ASW and SW co‐cultures were incubated for up to 100 days in optimal conditions for *Synechococcus* (as described earlier). We allowed triplicate flasks for each one of the eight time points analyzed, i.e., days 1, 3, 7, 14, 21, 32, 60 and 100. At each time point, *Synechococcus* cell abundance was monitored by flow cytometry (BD FACScan), while viable heterotrophs were counted by colony forming units on marine agar (Difco, France) as previously suggested (Christie‐Oleza *et al*., 2017).

For motility visualization, *R. pomeroyi* was grown in 10 ml of marine broth for 40 h after which cells were washed in filter‐sterilized autoclaved SW, re‐suspended in 10 ml of SW and further incubated for 4 days to starve the cells. Then, 100 µl of starved cells were added to 100 µl of ASW supplemented with 2.5 mM of (NH_4_)_2_SO_4_ and 4× the standard concentration of f2‐media vitamin mix. Succinate, glucose, yeast extract (Merck, Germany) and bacto peptone (Merck, Germany) were added at final concentrations, 0.005%, 0.01%, 0.02%, 0.05% and 0.1% (wt/vol), and incubated for 4 h before imaging cell motility using concave microscope slides under a 100× objective of a light microscope (Nikon Eclipse Ti) equipped with a widefield camera (Andor Zyla sCMOS). For each one of the conditions, 10 s videos were recorded from three different fields containing approximately 60 ± 10 cells and the number of motile cells was counted.

### Preparation of exoproteome samples

Triplicate 100 ml ASW and SW cultures were used for each time point. The exoproteomes contained in the culture milieu were collected after removing all cells via centrifugation at 4000 r.p.m. for 15 min at room temperature and further filtering the supernatant through 0.22 μm pore size filters (Millex‐GV; Millipore, Germany). A total of 40 and 80 ml of the supernatant of SW and ASW cultures, respectively, were used for trichloroacetic acid precipitation as described previously (Christie‐Oleza and Armengaud, 2010). The resulting protein pellets were dissolved in LDS loading buffer (Invitrogen, USA), and the equivalent of 20 ml of ASW cultures and 40 ml of SW cultures were loaded on a precast Tris‐Bis NuPAGE gel (Invitrogen, USA) using 1× MOPS solution (Invitrogen, USA) as the running buffer. SDS‐PAGE was performed for a short gel migration (5 mm). This allowed removing contaminants and purifying the polypeptides in the polyacrylamide gel.

### Trypsin in‐gel proteolysis and nanoLC‐MS/MS analysis

Polyacrylamide gel bands containing the exoproteome were excised and standard in‐gel reduction with dithiothreitol and alkylation with iodoacetamide were performed prior to trypsin (Roche, Switzerland) proteolysis (Christie‐Oleza and Armengaud, 2010). The resulting tryptic peptide mixture was extracted using 5% formic acid in 25% acetonitrile and concentrated at 40°C in a speed‐vac. For mass spectrometry, the samples were resuspended in 2.5% acetonitrile containing 0.05% trifluoroacetic acid and filtered using a 0.22 μm cellulose acetate spin column 16,000*g* for 5 min in order to eliminate undissolved aggregates. Sample were analyzed by means of nanoLC‐ESI‐MS/MS using an Ultimate 3000 LC system (Dionex‐LC Packings) coupled to an Orbitrap Fusion mass spectrometer (Thermo Scientific, USA) using a 60 min LC separation on a 25 cm column and settings as previously described (Christie‐Oleza *et al*., 2015b).

### Data analysis

Raw files were processed using the software package for shotgun proteomics MaxQuant version 1.5.5.1 (Cox and Mann, 2008) to identify and quantify protein using the UniProt databases of *Synechococcus* sp. WH7803 and *R. pomeroyi* DSS‐3. Samples were matched between runs. Other parameters were set by default. The peak intensities across the whole set of measurements were compared to obtain quantitative data for all the peptides in all the samples. Peak intensities and spectral counts assigned to each organism present in the co‐culture were compared (Supporting Information Table S1). The list of detected peptides and polypeptides is provided as Supporting Information Tables S2 and S3, respectively. The bioinformatic analysis pipeline was completed using the software Perseus version 1.5.5.3. Decoy and contaminants were removed. The relative protein abundance was obtained from the raw protein intensities from each sample after normalization to protein size and prior to converting to a logarithmic scale with base 2 (Murugaiyan *et al*., 2016). The missing values were imputed using the default parameters. The protein quantification and calculation of statistical significance were carried out using two‐sample Student's *t* test (*P* = 0.05) using a permutation‐based false discovery rate (*q* = 0.05). Protein categorization was based on KEGG annotations with manual curation using the Conserved Domain search tool from NCBI. Prediction of secreted proteins was carried out using the servers SignalP 4.1 (Petersen *et al*., 2011), SecretomeP 2.0 (Bendtsen *et al*., 2005), LipoP 1.1 (Juncker *et al*., 2003) and PSORTb (Yu *et al*., 2010).

## Supporting information

Additional Supporting Information may be found in the online version of this article at the publisher's web‐site.

**Table S1.** Spectral counts and mass intensities assigned to *Synechococcus* sp. WH7803 and *R. pomeroyi* DSS‐3 in each one of the mass spectrometry runs. Percentage assigned to each organism in the co‐culture is in brackets.

**Table S2.** List of peptides in the exoproteomes of *Synechococcus* sp. WH7803 and *R. pomeroyi* DSS‐3 co‐cultures detected by LC‐MS/MS.

**Table S3.** Raw list of polypeptides detected by LC–MS/MS in the exoproteomes of *Synechococcus* sp. WH7803 and *R. pomeroyi* DSS‐3 co‐cultures.

**Table S4.** (a) Protein categories and comparative exoproteomic analysis of *Synechococcus* sp. WH7803 proteins detected in SW co‐cultures with *R. pomeroyi* DSS‐3. (b) Protein categories and comparative exoproteomic analysis of *Synechococcus* sp. WH7803 proteins detected in ASW co‐cultures with *R. pomeroyi* DSS‐3.

**Table S5.** (a) Protein categories and comparative proteomic analysis of *R. pomeroyi* DSS‐3 proteins detected in SW co‐cultures with *Synechococcus* sp. WH7803. (b) Protein categories and comparative proteomic analysis of *R. pomeroyi* DSS‐3 proteins detected in ASW co‐cultures with *Synechococcus* sp. WH7803.

**Movie S1.** *R. pomeroyi* incubated in filter‐sterilized ASW obtained from a two‐week old *Synechococcus* culture.

**Movie S2.** *R. pomeroyi* incubated in ASW supplemented with ammonium and vitamins. Only Brownian motion is observed.

**Movie S3.** *R. pomeroyi* incubated in ASW supplemented with ammonium, vitamins and 0.02% peptone (wt/vol).
